# Different but complementary roles of action and gaze in action observation priming: Insights from eye- and motion-tracking measures

**DOI:** 10.3389/fpsyg.2015.00569

**Published:** 2015-05-05

**Authors:** Clément Letesson, Stéphane Grade, Martin G. Edwards

**Affiliations:** ^1^Psy-NAPS Group, Institut de Recherches en Sciences Psychologiques, Université Catholique de LouvainLouvain-la-Neuve, Belgium; ^2^Institute of Neuroscience, Université Catholique de LouvainLouvain-la-Neuve, Belgium

**Keywords:** mirror neurons, action observation, eye gaze, action priming, action prediction

## Abstract

Action priming following action observation is thought to be caused by the observed action kinematics being represented in the same brain areas as those used for action execution. But, action priming can also be explained by shared goal representations, with compatibility between observation of the agent’s gaze and the intended action of the observer. To assess the contribution of action kinematics and eye-gaze cues in the prediction of an agent’s action goal and action priming, participants observed actions where the availability of both cues was manipulated. Action observation was followed by action execution, and the congruency between the target of the agent’s and observer’s actions, and the congruency between the observed and executed action spatial location were manipulated. Eye movements were recorded during the observation phase, and the action priming was assessed using motion analysis. The results showed that the observation of gaze information influenced the observer’s prediction speed to attend to the target, and that observation of action kinematic information influenced the accuracy of these predictions. Motion analysis results showed that observed action cues alone primed both spatial incongruent and object congruent actions, consistent with the idea that the prime effect was driven by similarity between goals and kinematics. The observation of action and eye-gaze cues together induced a prime effect complementarily sensitive to object and spatial congruency. While observation of the agent’s action kinematics triggered an object-centered and kinematic-centered action representation, independently, the complementary observation of eye-gaze triggered a more fine-grained representation illustrating a specification of action kinematics toward the selected goal. Even though both cues differentially contributed to action priming, their complementary integration led to a more refined pattern of action priming.

## Introduction

Making sense of the behaviors of others and predicting the likely outcome of their actions is an essential component of interactive behavior (Wilson and Knoblich, 2005), thought to rely on common action observation and execution neural processes (e.g., the mirror neuron system, MNS; Rizzolatti and Craighero, 2004). These overlapping processes between observation and execution allow for the observation of a specific action to activate the observer’s motor system. One consequence of these shared processes is that the observation of action can moderate action execution for different action components, including action speed or timing (Edwards et al., 2003), action force (Salama et al., 2011) and action spatial trajectory (Hardwick and Edwards, 2011). This facilitation, known as the action priming effect, demonstrates that the shared neural processes between action observation and execution must encode precise information about the perceived action.

Recently, Wilson and Knoblich (2005) suggested that action observation neural processes incorporate predictive cognition. Observers use their own motor system to model (or represent) observed actions, allowing for the computation and prediction of an agent’s behavior and unfolding action. In this sense, the neural processes are not simply activated in a bottom–up fashion by the mere observation of others’ actions, but rather in anticipation to them. For example, Kilner et al. (2004) presented participants with video clips of either a stationary hand flanked by an object or a moving hand grasping an object. The color of the object indicated the type of video stimuli presented; either the hand would remain stationary, or whether it would move to grasp the object. The results showed that the object color associated with the moving grasp action condition caused predictive motor neural activity in anticipation of the initiation of the moving hand stimuli, whereas there was no such motor neural activity in anticipation of the static hand stimuli. A similar effect was reported by Umiltà et al. (2001). They reported results from a set of motor neurons that was active both for the observation of a fully visible reach and grasp action to an object, and also for the observation of a similar action where part of the reach and the grasp was occluded from vision by a screen (though the observer knew that there was a target object behind the screen). In this case, an early anticipation of the action goal must have been computed. The observer must have somehow relied on other visual information to extract relevant cues regarding the presence of the occluded action and target, and this caused predictive motor neural activation.

One might assume that the observation of the unfolding action could be sufficient for the observer to anticipate the action goal. However, Flanagan and Johansson (2003) showed that participants paid very little attention to the unfolding action, but instead, they implemented proactive eye movements similar to those used during actual action execution, where eye-gaze was anticipatively directed to the end-point or the goal of the action (see Rotman et al., 2006 for similar findings). This attentive pattern was explained as a procedure to provide visual feedback about the ongoing action execution relative to the target object or final action goal, and that any errors in the action trajectory could be anticipated and perceived to provide information for correction (Land and Furneaux, 1997; Gesierich et al., 2008). Indeed, during visually guided actions, there is little doubt that proactive gaze behaviors are essential for correct planning and coherent control of the executed motor program (Johansson et al., 2001).

The finding that observers use predictive eye-gaze patterns during action observation suggests that information from different visual sources must be obtained in order to infer the intended action goal. These visual cues could emerge from an early analysis of the agent’s behaviors before the observed action is fully executed. In this sense, Ambrosini et al. (2011) measured how fast and how accurately participants were able to anticipatively gaze at an agent’s intended action target. Participants were asked to observe several types of reach-to-grasp actions while their eye movements were recorded. The observed actions could be directed to one of two different sized objects (small versus large), and the agent could either correctly pre-shape their hand to the target object (e.g., precision grip versus whole hand grip to the small and large sized objects respectively) or the agent showed no pre-shaping of their hand when acting to the objects (e.g., closed fist). The results showed that the hand pre-shaping condition caused participants to gaze at the correct target object quicker and more accurately than for the no hand pre-shaping condition. This suggests that hand information during the action observation provided a reliable cue to allow an early prediction of the intended target or action goal.

Hand-shape motoric cues are not the only source of information allowing for the prediction of the agent’s action goal. In action execution, we normally first gaze toward an object that we intend to interact with, before actually acting upon the object (Johansson et al., 2001; Land and Hayhoe, 2001). The information that the observer could glean from the agent’s gaze would constitute a reliable cue to predict object interaction intention (Becchio et al., 2008). Indeed, many studies have already examined the automatic tendency of the observer to orient their gaze to the same location as an agent’s perceived gaze (Driver et al., 1999; Langton et al., 2000). Similarly, Castiello (2003) showed that the observation of another person’s gaze toward a target object, as well as an actual action, reliably primed action execution. Further, Pierno et al. (2006) used functional magnetic resonance imaging (fMRI) to measure brain activity when participants observed video clips of a human model either reaching and grasping a target object or gazing at an object. The contrast between these two conditions and a control condition (in which the agent stood behind the object and performed no action or gaze) revealed similar profiles of brain activity. This suggests that the two types of information might be represented in a common motor code, and that either information could be sufficient to prime action execution. However, currently it remains unclear how action and gaze information interact during action observation, and whether the different types of information moderate the observer’s executed gaze patterns and subsequent action responses.

The aim of the present study was twofold. First, we aimed to provide evidence for attentive and predictive eye-gaze behavior during action observation by measuring the observer’s eye movements to different specified regions of interest (ROI), and by measuring the speed and accuracy of anticipatory gaze relative to manipulations of the agent’s gaze and action kinematics to a target object. The interest of the latter analysis was to determine which of the gaze or action visual cue information would be selected when the two types of information were manipulated in the visual scene. We aimed to replicate previous studies that have investigated the predictive functioning of action observation processes (Rotman et al., 2006; Webb et al., 2010; Ambrosini et al., 2011, 2012) and in addition, demonstrate that the agent’s gaze provides early cues that indicate an intention to grasp a particular target object. Additionally, as observers have been shown to be efficient at extracting action intention information from both gaze cuing and observed actions (Sartori et al., 2011), we expected that, in the absence of the agent’s gaze, the participants would orient their attention to the ongoing action as a secondary source of information. The second aim of the study was to better understand the different and complementary effects that gaze and action cues could have on the action priming effect. Recent studies showed that observed actions can be encoded in terms of their goal (Rizzolatti and Craighero, 2004), but also that goal representations of observed actions can be accompanied with more specific information regarding action kinematics, such as action trajectories (Griffiths and Tipper, 2009; Hardwick and Edwards, 2011). We therefore expected that the observation of both gaze-object and hand-object interactions would moderate subsequent action execution kinematics. However, in the current scientific literature, it remains unclear if these cue-induced priming effects are driven by a similarity of goals and/or trajectories between the observed and the executed actions. Therefore, we assessed the contribution of goal information by manipulating the congruency of the target objects during action observation and action execution, and further, we investigated the contribution of kinematics information through the manipulation of spatial congruency between the observed and executed actions.

## Materials and Methods

### Participants

We tested a total of 22 persons, though three participants were excluded because of corruptions in their data recording (i.e., recording failures causing unusable data) and were not analyzed any further. The mean age of the remaining 19 participants was 22.1 years (range: 2.3 years), all were right-handed (self-reported) and had normal or corrected-to-normal visual acuity. All participants gave their informed consent to take part in the study and they were remunerated for their participation. The Université Catholique de Louvain, Faculty of Psychology Ethics Commission approved the experiment.

### Apparatus and Stimuli

To record participants’ eye movements, we used the Eyelink 1000 desktop mounted eye tracker (SR Research, Canada; sampling rate of 1000 Hz; average accuracy range 0.25–0.5∘, gaze tracking range of 32∘ horizontally and 25∘ vertically). Participants sat at a distance of 60 cm from the eye tracker camera and head movements were prevented by using a chin and forehead stabilizer. At the beginning of each trial block, a standard 9-point protocol was used to calibrate the participant’s eye-gaze position to a display screen using standard Eye-Link software. This allowed the computation of the actual gaze position on the screen. To record participants’ hand actions, we used the Polhemus Liberty electromagnetic 3D motion tracker (Polhemus Incorporated, Colchester, Vermont; sampling rate of 240 Hz, accuracy 0.076 cm for position and 0.15∘ for orientation). Sensors were attached to two target objects, and the participant’s wrist, thumb, and index finger using adhesive tape and a flexible wrist splint. The kinematic data were analyzed oﬄine and the dependent measurements were extracted from the 3D XYZ coordinates.

**FIGURE 1:**
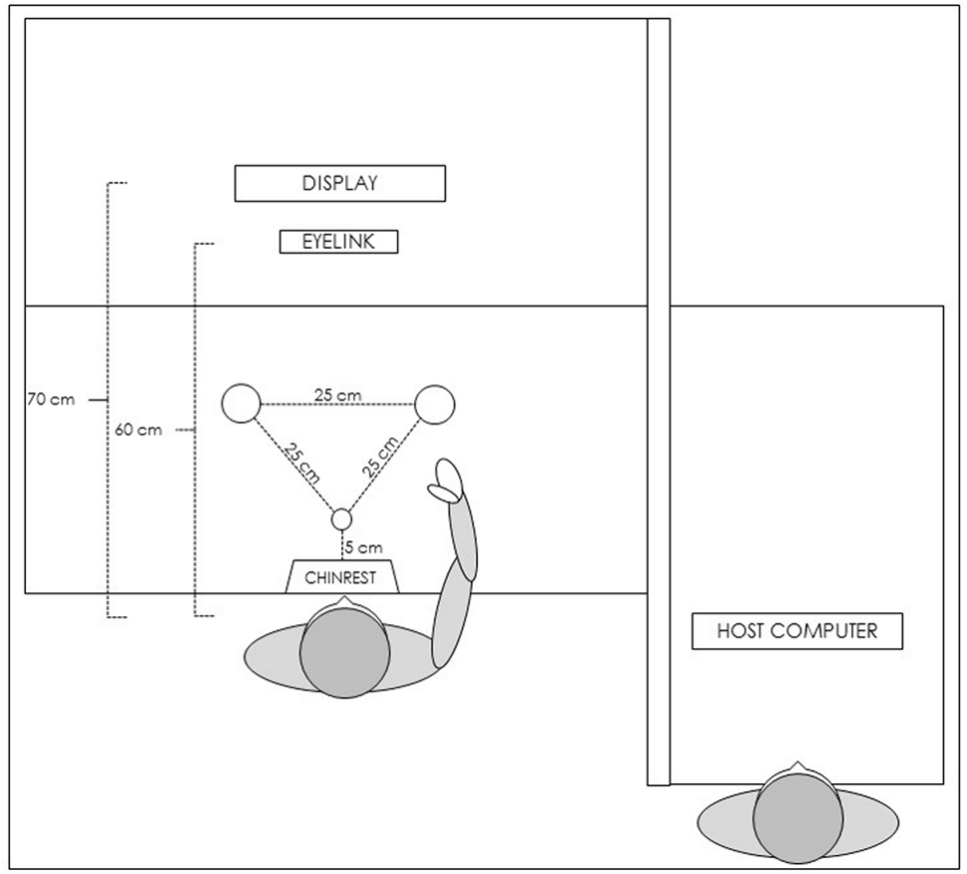
**Schematic representation of the experimental setup**.

The laboratory arrangement consisted of three wooden tables (120 × 80 cm) creating an L-shaped workspace (see **Figure 1**). Both the participant and the experimenter faced the same direction, with the participant to the left of the experimenter. The experimenter was positioned (offset) behind the participant allowing a view of the participants’ workspace/screen without distracting them. We further shielded distractions by placing a wooden panel between the tables (occluding all of the computer equipment that we used for the eye-tracker and motion tracker recordings). On the participant’s table, the chin and forehead stabilizer was placed centrally, 5 cm from the table edge. A computer screen (LCD; resolution 1080 × 1920; refresh rate 60 Hz) was placed 70 cm from the chin and forehead stabilizer, and was used to display visual stimuli for the experiment. The visual stimuli were presented using E-Prime (v2.0.8.90 PRO; Schneider et al., 2002).

**FIGURE 2:**
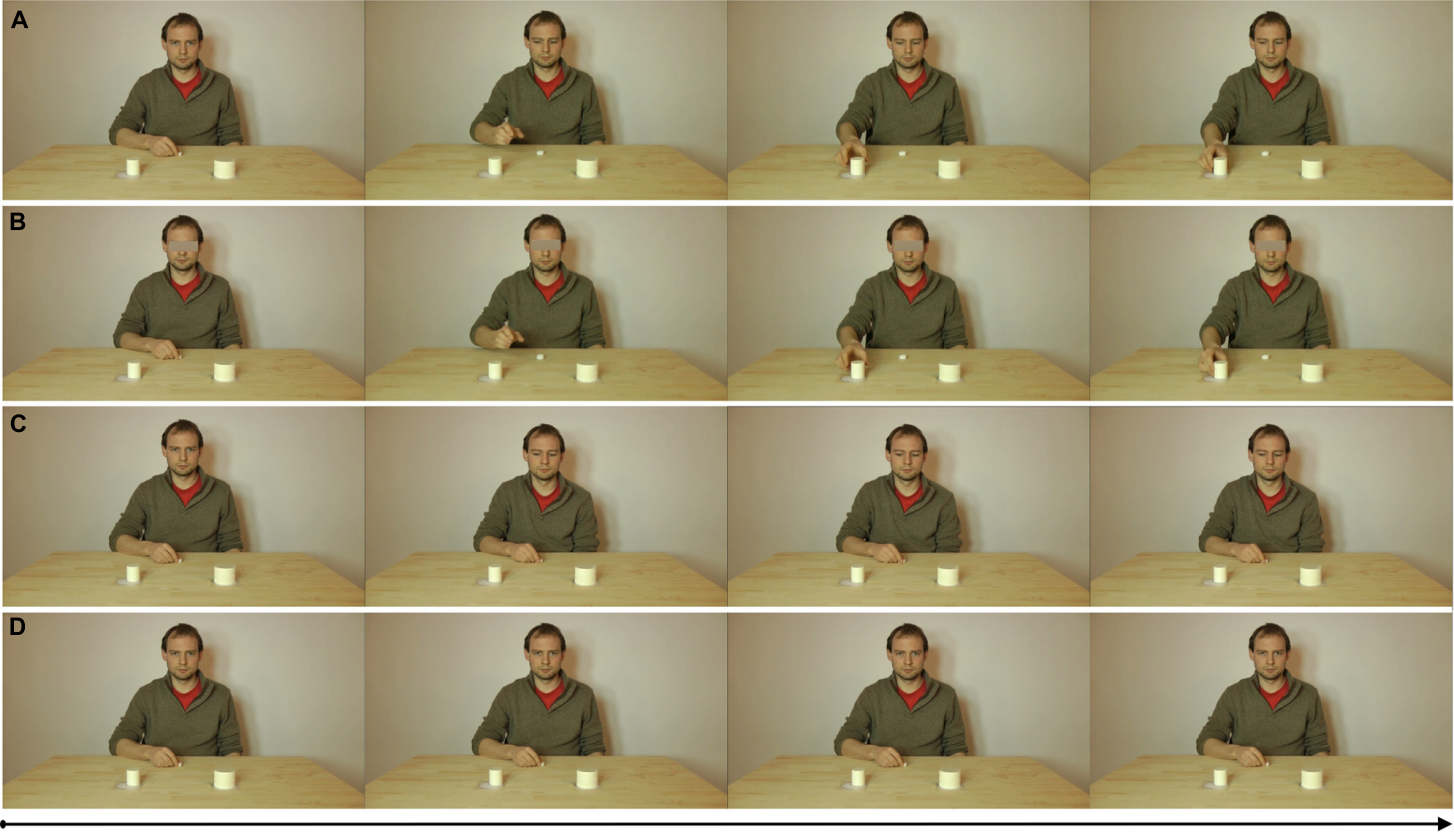
**Examples of observation video clips from each experimental condition**. From top to bottom: conditions **(A)** FULL, **(B)** ACTION, **(C)** GAZE and **(D)** CONTROL. The videos were presented on a HD screen (1920^∗^1080 pixel). The actor’s eyes area occupied a 3 cm screen area and participants viewed the screen at a distance of 70 cm (equivalent to ∼2.45∘ of visual angle and equivalent to observing a real-sized human at a distance of ∼220 cm).

The experiment involved the use of two types of stimuli (visual and physical). The visual stimuli consisted of video clips presented on the computer screen depicting reach-to-grasp actions (AVI format, 25 fps, 1920 × 1080 pixels). The video clips consisted of a male agent sitting at a wooden table and looking into the camera, with his right hand holding a reference object (∅: 2 cm) in front of him. A small and a large object were also presented (∅: 4 and 7 cm), 25 cm in front of the agent, one on the left and the other on the right of the agent’s sagittal midline (25 cm apart, and their position counterbalanced). In the videos, we manipulated the availability of the agent’s gaze and action (see **Figure 2**). In the gaze and action (FULL) condition, the video started by showing the agent positioned facing the participant, looking into the camera lens, and a small and a large object presented on the table, symmetrical and of equal distance to the sagittal axis. Next, the agent directed his gaze toward either the small or the large object, and then he executed a reach-to-grasp action to the object that he gazed toward. In the gaze only condition (GAZE), the video again started by showing the agent looking into the camera lens (etc.). Next, he directed his gaze toward one of the two objects (as in the previous condition), but this time, no action was executed. Therefore, the agent’s gaze direction was the only available cue indicating the target object. In the action only condition (ACTION), we placed a mask on the eyes area of the agent. The video started by showing the agent with the eye mask (preventing gaze cues; but with all other aspects of the video matched to the FULL condition). The only cue was of the agent executing a reach-to-grasp action to the object. The final condition was the no gaze and no action condition (CONTROL). In this condition, neither eye-gaze information nor action information was presented. The agent remained still throughout the video. For each of the experimental conditions (FULL, GAZE, ACTION), there were four different types of videos that balanced the size and position of the target objects (small left, small right, large left, large right). In the control condition (CONTROL), there were only two different videos (small object left and large object right versus large object left and small object right). The videos were matched in length (4500 ms) and each video was presented eight times across two blocks of trials (with a total of 112 trials per participant).

The physical stimuli were presented in the participants’ physical workspace. On the participant’s sagittal axis, we placed a starting action reference object (∅: 2 cm) positioned 5 cm further from the chin and forehead stabilizer. We also placed a small and a large round object (∅: 4 and 7 cm; the same objects as those presented in the visual stimuli conditions), 25 cm from the reference object, and symmetrical to the participant’s sagittal axis (with the edge of the objects 12.5 cm from the sagittal midline). The object positions were counterbalanced. At the beginning of each trial, the participant was asked to hold the starting action reference object with a right hand light grip (providing a common action origin point for the comparison between responses).

### Design and Procedure

Each trial started with the presentation of a fixation cross in the center of the screen and the participant was instructed to look at the cross. This ensured that each participant would start observing the video sequences from the same origin point, allowing for a comparison between gaze paths. The fixation cross was also used as a drift check to verify and confirm the reliability of the eye-gaze calibration. The fixation cross was displayed until the experimenter manually confirmed that the participants’ gaze was fixed to the cross position. As soon as participants’ gaze position was confirmed, one of the video clips was randomly presented. The participant was instructed to observe and attend to the video carefully, as they would be required to make an action in subsequent part of the trial. At the end of the video, a sound was presented that indicated the size of the object that the participant had to grasp during the final execution part of the trial. A low-tone indicated that the participant would have to grasp the large physical object and a high-tone indicated that the participant would have to grasp the small physical object. The participants were instructed that when they heard the sound, they had to reach, grasp and lift the target object in a natural manner (“as if you were reaching and grasping your cup of tea”), each time, initiating the action from the action reference object.

### Data Analyses

The results were analyzed for both eye-tracking and motion-tracking measures. For the post-tests analyses, a Bonferroni correction was applied. For the data analyses, we separated the visual scene in the video clips into five ROI that were slightly larger than the part of interest in the visual scene (compensating for any variance in the eye-tracking data). The regions selected corresponded to the fixation cross, the agent’s head, the agent’s hand and the two target objects (the left object and the right object). Participant’s eye-gaze to each ROI was considered in the analyses and any eye-gaze outside of the ROI was not included in the data analyses. The grasped or gazed object was denominated as “TARGET” and the non-target object as “NON-TARGET” (irrespective of the object size or location).

We used three dependent variables to analyze the eye-tracking data. The first was the proportion of total fixation time spent in each ROI (with ROI added as an independent variable). The aim of this analysis was to investigate how manipulations to gaze and action cues moderated the participants’ attention to the manipulated bodily cues. Therefore, we analyzed whether the observations conditions induced different attentional profiles to specific ROIs. For this analysis, we only included trials where the participant started the trial by fixating the cross (98% valid trials). The second dependent variable was prediction speed; derived from the time elapsed between the participant’s first fixation to the target object ROI and either (i) the time when the agent’s gaze was directed to the target object (gaze-based index) or (ii) the time when the agent’s hand reached the target object (action-based index). The prediction speed indexes were specific to the conditions that manipulated these cues; with planned *t*-test contrasts for the gaze-based index comparing GAZE versus FULL conditions, and the action-based index comparing ACTION versus FULL conditions; no comparisons were possible between the CONTROL or between the GAZE and ACTION conditions. Therefore, the gaze-based index and the action-based index allowed us to measure respectively the contribution of action and gaze information to prediction speed. Here, only trials where participants correctly oriented their gaze toward the target of the agent’s attention or action were included. We excluded trials in which participants oriented their gaze toward a target before any gaze or action cues were presented in the video clip (84.5% valid trials). The third dependent variable was prediction accuracy that was defined as the proportion of trials where the participant correctly oriented their eye (attention) toward the target of the agent’s attention or action. This variable measured the efficiency of our experimental manipulations in producing correct predictions. The same contrasts as those used for prediction speed were applied to this variable.

For the motion-tracking analyses, we determined two levels of congruency between the observation and the execution conditions: (i) ‘object congruency’ irrespective of spatial location (congruent: observation of action to the same sized object as that grasped in the execution condition; small–small or large–large versus incongruent: observation of action to a different sized object as that grasped in the execution condition; small–large or large–small); and (ii) ‘spatial congruency’ irrespective of object size (congruent: the same egocentric spatial location for the agent and observer in the observed and execution conditions; agent reaching to their left and participant reaching to their right or agent reaching to their right and participant reaching to their left versus incongruent: different egocentric spatial action location for the agent and observer in the observed and execution conditions; agent reaching to their left and participant reaching to their left or agent reaching to their right and participant reaching to their right). The prime effect was measured with three dependent variables: reaction time (ms; with action initiation being defined as the time when the hand velocity reached 50 mm/s for two successive frames), time to peak velocity (ms), and time to peak grasp aperture (ms). As the aim of the kinematics analysis was to understand how the cue-induced priming effects were differentially sensitive to object and spatial congruency, we defined a priori analyses to check how these two variables would interact throughout each video condition. Three-way interactions were decomposed by evaluating how object congruency and spatial congruency interacted in each video condition independently. If second order two-way interactions were significant, we performed multiple comparisons between object and spatial congruency. The rationale behind the present statistical approach is similar to that described by Howell and Lacroix (2012) for decomposing three-way interactions.

## Results

### Eye-Tracking Analyses

**FIGURE 3:**
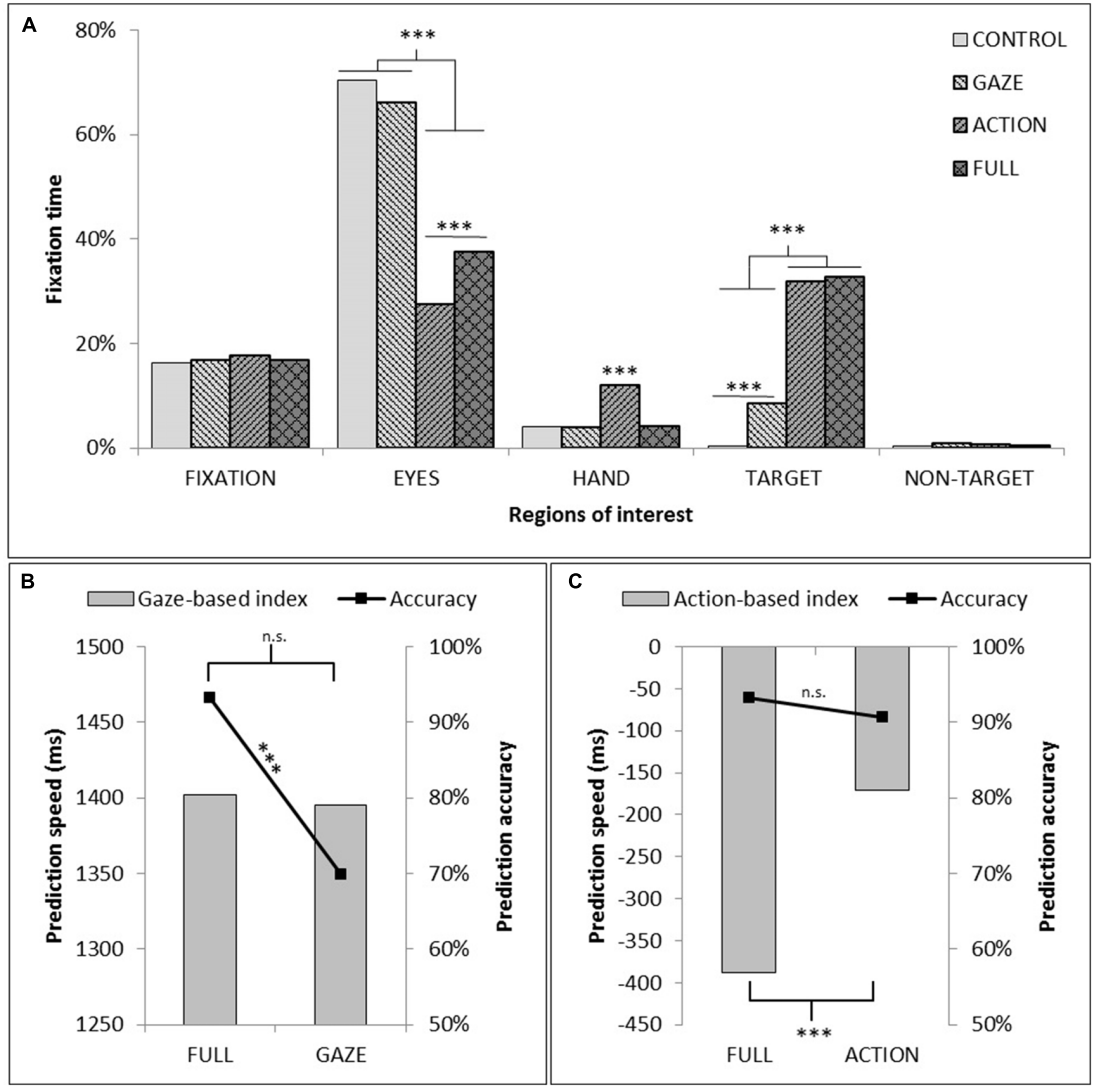
**(A)** Proportion of total fixation time within each of the five ROIs as a function of observation conditions (In the CONTROL condition, as no specific object could be reported as ‘TARGET’ or ‘NON-TARGET,’ the average fixation time on both objects was plotted). **(B)** Measure of the contribution of action information to prediction speed and prediction accuracy through the comparison of GAZE and FULL conditions. **(C)** Measure of the contribution of gaze information to prediction speed and prediction accuracy through the comparison of ACTION and FULL conditions. The asterisks indicate a significant difference between experimental conditions (^∗^*p* < 0.05; ^∗∗^*p* < 0.01; ^∗∗∗^*p* < 0.005).

The repeated measures ANOVA for the proportion of total fixation time spent in the ROIs compared within-participant factors of video conditions (FULL, GAZE, ACTION, CONTROL) and ROIs (fixation, the agent’s head, the agent’s hand, the target and the non-target). We found significant main effects for the video conditions [*F*(3,54) = 3.98, *p* < 0.05, $\eta_{p}^{2}$ = 0.18] and ROIs [*F*(4,72) = 282.18 *p* < 0.001, $\eta_{p}^{2}$ = 0.94], and a significant interaction [*F*(12,216) = 106.17, *p* < 0.001, $\eta_{p}^{2}$ = 0.85]. The pairwise comparisons of the main effects showed that the proportion of total fixation time within the five ROIs was significantly lower for the ACTION than for the FULL and GAZE video conditions. Also, the proportion of total fixation time was significantly different for each ROI, except for the target object and the fixation areas. The agent’s head was fixated significantly longer than any other ROI, and the target object was fixated longer than the non-target object. We decomposed the interaction by evaluating each ROI separately. This showed significant observation condition effects for the ROIs of the agent’s head [*F*(3,54) = 90.13, *p* < 0.001, $\eta_{p}^{2}$ = 0.83; with GAZE and CONTROL conditions eliciting longer fixation time compared to ACTION or FULL conditions], the agent’s hand [*F*(3,54) = 29.25, *p* < 0.001, $\eta_{p}^{2}$ = 0.62; with ACTION significantly different from the other conditions], and the target [*F*(3,54) = 167.32, *p* < 0.001, $\eta_{p}^{2}$ = 0.9; with both CONTROL and GAZE being different from each other and the other two conditions]. There was a significant effect for the non-target ROI [*F*(3,54) = 3.19, *p* < 0.05, $\eta_{p}^{2}$ = 0.15; though the pairwise comparisons did not highlight any significant differences]. See **Figure 3A**.

The analysis of prediction speed and accuracy dependent variables compared the FULL condition with each of the manipulated GAZE or ACTION conditions using planned *t*-test and evaluated a gaze-based index and an action-based index (time between participant’s target fixation and agent’s eye-gaze or time between participant’s target fixation and the agent’s hand action to the target). Analysis of the gaze-based index showed no significant effect for prediction speed [*t*(18) = 0.85, *p* = 0.4, *d* = 0.19], but there was a significant effect of prediction accuracy [*t*(18) = -4.5, *p* < 0.001, *d* = 1.08]. The participant fixated to the correct target more frequently when both the agent’s eye-gaze and action cue information was presented compared to the videos with the gaze cue alone, indicating that the presence of action information contributed to the correct orientation of participant’s attention to the target object. Analysis of the action-based index showed a significant effect of prediction speed [*t*(18) = -8.7, *p* < 0.001, *d* = 2], but no effect of prediction accuracy [*t*(18) = -1.16, *p* = 0.26, *d* = 0.26]. The participant fixated to the target faster when both the agent’s eye-gaze and action cue information was presented compared to the videos with the action cue alone, indicating that the processing of gaze information contributed to prediction speed (see **Figure 3**).

### Motion-Tracking Analyses^1^

**FIGURE 4:**
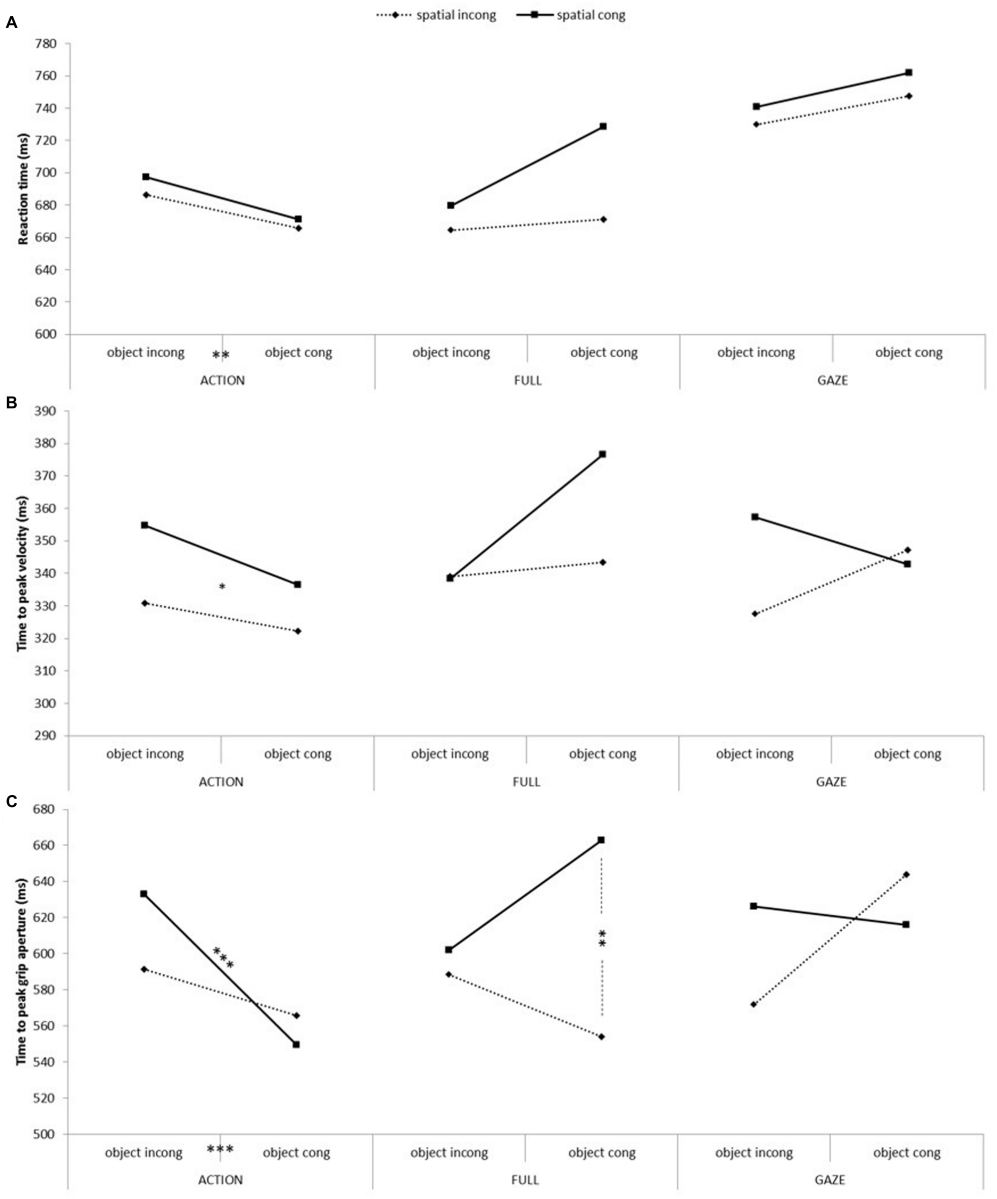
**Participants’ action measures as a function of conditions, object congruency and spatial congruency**. From top to bottom: **(A)** Reaction time, **(B)** time to peak velocity and **(C)** time to peak grip aperture. The asterisks indicate a significant difference between experimental conditions (^∗^*p* < 0.05; ^∗∗^*p* < 0.01; ^∗∗∗^*p* < 0.005).

To assess ^1^ the action priming effect, we tested the independent variables of video conditions (FULL, GAZE, ACTION), object congruency, and spatial congruency using repeated measures ANOVAs on the three dependent variables of reaction time, time to peak velocity and time to peak grip aperture. CONTROL condition could not be included in the model as it did not vary for the spatial and object congruency independent variables. All of the results for this section are presented in **Figure 4**.

The analysis of the participants’ reaction time showed a significant main effect of video condition [F(2,36) = 47.37, *p* < 0.001, $\eta_{p}^{2}$ = 0.72], with pairwise comparisons showing that the actions executed after the observation of the ACTION or FULL video conditions were initiated significantly faster than after the observation of the GAZE video condition. There was also a significant main effect of spatial congruency [*F*(1,18) = 5.87, *p* < 0.05, $\eta_{p}^{2}$ = 0.25], with spatial incongruent actions being initiated faster than spatial congruent actions. Finally, the results showed a significant interaction between video condition and object congruency [*F*(2,36) = 5.87, *p* < 0.01, $\eta_{p}^{2}$ = 0.25], showing that object congruent trials were initiated faster than object incongruent trials in the ACTION condition only [*t*(18) = 2.86, *p* < 0.05, *d* = 0.66].

The analysis of time to peak velocity showed a significant main effect of spatial congruency [*F*(1,18) = 5.16, *p* < 0.05, $\eta_{p}^{2}$ = 0.22], showing that the observation of spatial incongruent action caused a faster time to peak velocity compared to spatial congruent actions. There was also a significant three-way interaction [*F*(2,36) = 3.36, *p* < 0.05, $\eta_{p}^{2}$ = 0.16] that was decomposed with an ANOVA for each video condition. These analyses only showed a significant spatial congruency effect for the ACTION video condition [*F*(1,18) = 5.78, *p* < 0.05, $\eta_{p}^{2}$ = 0.24], with spatial incongruent trials reaching their peak velocity quicker than the congruent ones. None of the other contrasts were significant once corrected.

There was a significant effect for time to peak grasp aperture for spatial congruency [*F*(1,18) = 5.43, *p* < 0.05, $\eta_{p}^{2}$ = 0.23], with the peak grip aperture being reached quicker for spatial incongruent than congruent actions. There was a significant interaction between video conditions and spatial congruency [*F*(2,36) = 3.27, *p* < 0.05, $\eta_{p}^{2}$ = 0.15], showing a faster time to peak grasp aperture in the spatial incongruent compared to congruent actions in the FULL video condition only [*t*(18) = 2.95, *p* < 0.01, *d* = 0.67]. There was also an interaction between video conditions and object congruency [*F*(2,36) = 5.7, *p* < 0.01, $\eta_{p}^{2}$ = 0.24] showing a significantly faster time to peak grasp aperture for the object congruent than incongruent trials in the ACTION condition [*t*(18) = 3.87, *p* < 0.001, *d* = 0.88]. Finally, there was a three-way interaction between all of the independent variables [*F*(2,36) = 7.7, *p* < 0.01, $\eta_{p}^{2}$ = 0.30] that was analyzed using ANOVAs for each video condition separately. Significant two-way interactions between spatial and object congruency were found for the FULL and GAZE video conditions [*F*(1,18) = 5.27, *p* < 0.05, $\eta_{p}^{2}$ = 0.23; *F*(1,18) = 7.23, *p* < 0.05, $\eta_{p}^{2}$ = 0.29; respectively]. Based on our hypothesis, paired-samples *t*-tests defined *a priori* contrasted spatial and object congruent versus incongruent conditions. For the FULL video condition, there was only a significant effect of spatial congruency when the objects were congruent [*t*(18) = 3.28, *p* < 0.01, *d* = 0.75], showing that spatial incongruent actions reached their peak grasp aperture faster than the spatial congruent actions. For the GAZE condition, none of the pairwise comparisons reached significance.

## Discussion

The aim of the present study was firstly to evaluate foveal attention and predictive eye-gaze behaviors during action observation and, secondly, to better understand the complementary effects that eye-gaze and action cues have on the action observation priming. To evaluate natural foveal attention during the observation conditions, we determined how manipulations to gaze and action cues moderated the participants’ attention to specific ROIs. Overall, this showed that participants spent more time fixating the head area of the agent in all of the observation conditions, although it was fixated more in the GAZE and CONTROL conditions compared to the ACTION and FULL observation conditions. In addition, the hand area of the agent was fixated more when only ACTION information was presented compared to the other observation conditions, and participants looked at the target for longer in the FULL and ACTION observation conditions relative to the GAZE and CONTROL conditions.

We also measured the differential contribution of the agent’s gaze and action information cues on the participant’s action prediction. We proposed that particular cues might facilitate the participant’s prediction to selectively attend to the correct target. The results showed that the speed at which the participants correctly oriented their attention to the target of an observed action was influenced by the availability of eye-gaze information, whereas manipulating action information influenced the accuracy of the predictions. Interestingly, the combined availability of action and gaze cues provided the most reliable prediction cues for both prediction speed and accuracy. These results show that when observing a human agent performing a goal-directed action, participants appeared to prioritize attention to the agent’s eyes, and that information from both the agent’s eye-gaze and action cues were important for predicting the target to which the agent intended to act toward (the goal of the observed action). These findings are consistent with other studies showing a tendency for participants to attend to agent’s eye-gaze and looking direction (Senju and Hasegawa, 2005; Conty et al., 2006), an effect that is perhaps not surprising given the use of eye contact to establish communicative links between individuals (Farroni et al., 2002). In the case of action observation, the observer could glean information about the agent’s intentions through the establishment of joint attention (Driver et al., 1999). When performing actions, the agent usually attends to the object that they intend to act toward (Johansson et al., 2001; Land and Hayhoe, 2001) and therefore, this information appears to constitute a reliable source of predictive information.

Interestingly, there was proportionally very little attention allocated to the hand region during the observation of video clips during the FULL condition. However, observation of the agent’s eye-gaze and action cues during the FULL condition resulted in greater prediction accuracy compared to observation of only the agent’s eye-gaze cues during the GAZE condition. This suggests that the observation of hand trajectories must use peripheral vision, perhaps serving to reduce the ambiguity regarding the predicted action goal determined by the agent’s eye-gaze cues, and contributing to increased prediction accuracy. These findings support and extend previous findings by Webb et al. (2010) who presented participants with video clips depicting one of two human agents performing reach-to-grasp actions to one of three different targets aligned horizontally. Participants were asked to observe the videos and determine the agent and the to-be-grasped object. Before each video, the identity of the agent or the target was unknown to the participant. The results showed that agent’s eye-gaze direction and hand trajectory information were important in guiding the observer’s gaze to the correct target. Our data add to these results by evaluating the relative contribution of both action and gaze cues in guiding the observer’s gaze behaviors, and further, by showing that direct foveal attention to the observed hand trajectories was not necessary to cause the prediction effects, suggesting that action trajectories must have been attended to with peripheral vision.

The discrepancies between action and gaze information processing during action observation could somehow explain the trade-off between prediction speed and prediction accuracy. On the one hand, when no action information was available, the quick processing of the agent’s gaze was made at the expense of accuracy, where perhaps an insufficient amount of information regarding the goal of the action had not yet been gathered. On the contrary, the absence of gaze information forced the observers to gather more information about the intended goal of the action from early motor information (hence the hand ROI was fixated for longer in the ACTION condition compared to other conditions). As it is less obvious for the observers to rule out the intended goal from the agent’s action, they had to extrapolate the most likely target from the early trajectory of the agent’s hand. Two possible targets were available in our design. Therefore, for the observers to make a correct prediction only based on the observation of the agent’s hand trajectory, more time was needed to exclude the alternative object.

Flanagan and Johansson (2003) suggested that eye movements during action observation were proactive rather than reactive. Our results provide further support for this claim, and give insights into how different cues are processed together in order to provide reliable predictive cues about ongoing actions. In everyday life situations, the targets of our actions are not fully predictable to the observer. Our actions can be oriented to a target presented with multiple other objects, differing in sizes, colors, or even locations. During visually guided action execution, the agent must extract the various features from the selected target object and position in order to grasp the object successfully. For example, the agent typically will pre-shape their hand to the size of the intended target object, with a slight over-grasp allowing for an efficient grip placement on the object (Jeannerod et al., 1995; Eastough and Edwards, 2007), and they will monitor the position of other objects in proximity to the target, making critical kinematic modifications to avoid the obstacle positions. This necessity of visual inputs for action control legitimates the anticipative nature of agent’s eye movements during action execution (Johansson et al., 2001). Similar mechanisms were hypothesized during action observation conditions, as the human motor system is also involved in processes helping us to perceive the action of others (Wilson and Knoblich, 2005). Bach et al. (2011) provided evidence that the motor representations elicited during action observation were bi-determined. Not only did they match the observed actions, but they also reflected the proprieties of the goal objects that they were directed to. Accordingly, It has already been shown that observers are efficient in predicting future hand-object interactions by relying on hand pre-shaping cues (Ambrosini et al., 2011). Along the same lines, gaze cuing toward an object has been shown to provide a consistent indicator of a future interaction with that object, allowing the observers to predict the short term course of ongoing actions (Pierno et al., 2006). Our data take these findings one-step further by highlighting reliable and successful identification of the intended goal object from the processing of both the agent’s action and gaze cues compared to each cue in isolation. Action and gaze cues provided different, but complementary advantages for action prediction, indicating the implementation of different observation strategies depending on the nature of the information available.

The second aim of this study was to better understand the complementary effects that gaze and action cues could have on subsequent action execution (i.e., the action priming effect). By modifying the cues during the observation conditions, we evaluated the effects of spatial and object congruency on subsequent action performance. We found that participants responded with a faster reaction time in actions executed after the observation of the ACTION or FULL video conditions, than after the observation of the GAZE video condition (without action information). This suggests that action information more than gaze information contributed to the action priming effect, suggesting that the action cue had an impact on motor planning processes. An alternative suggestion though could be that the slower reaction time to the gaze cue relative to the other cues might have been a consequence of the lower rate of accurate eye-gaze target prediction during observation. Reaction time was also moderated for spatial congruency, showing that spatial incongruent actions were initiated faster than spatial congruent actions. This effect suggests that the observation of action was represented in a frame of reference centered on the observed agent; thus, when the participant observed a right hand action to a target object that was on the right of the agent (left of the participant), action was primed when the participant made a right hand action to a target on the right of the participant (the spatially incongruent target). This suggests priming between the observation and execution of action kinematics (the agent’s right hand action primed the observer’s right hand action; see Hardwick and Edwards, 2011 for similar priming of kinematic trajectories). This same effect was found for the dependent variables of time to peak velocity and time to peak grasp aperture. Further interaction analyses showed that the observation of the FULL condition (with gaze and action cues) compared to GAZE and ACTION conditions (with only one cue) caused participants to have a faster time to peak grasp aperture for the spatial incongruent compared to congruent actions, therefore replicating the main effect.

Counter evidence to the kinematic priming effects discussed above was shown for the interaction analyses of the ACTION condition (where no gaze information was presented). Reaction time and time to peak grasp aperture were earlier for congruent than incongruent target objects, supporting the idea of priming driven by common goals. The combined presentation of action and gaze cues (in the FULL condition) induced a more refined pattern of priming, sensitive to modulations of both object congruency and spatial congruency. The peak grip aperture was earlier for spatial incongruent actions (kinematic congruent) and later for spatially congruent actions (kinematic incongruent), only when the objects were congruent (similarity of goals). It is worth mentioning here that faster time to peak grip aperture is usually linked to a longer deceleration phase, allowing for a better control over the end-phase of the action and to adapt the hand to the state of the target (Jeannerod, 1994). This suggests that in the present findings, information regarding action goals and kinematic trajectories were important for the prime effect to appear and this probably improved grasp performance.

Supporting both the notion of a goal-driven priming and a kinematic priming, these data shed important light on the information extracted and represented during action observation. There is no doubt that the notion of goal is important in executed and observed actions, as shown by the goal-coding preferences of the motor system (Rizzolatti et al., 1996; Umiltà et al., 2001; Fogassi et al., 2005). The extraction of goal information from observed actions has been attributed to mirror neuron system function, where action goals are understood through the observed action resonating with the observer’s own motor system. This mechanism has been proposed to allow for the prediction of the action goal based on simulation and perhaps prior experience of action execution (see Rizzolatti and Craighero, 2004; Rizzolatti and Sinigaglia, 2010 for reviews).

In the scientific literature to date, there has been little investigation to understand whether the presence of eye-gaze and action information during action observation differentially moderate the action priming effect. For example, in Edwards et al. (2003), both eye-gaze and action cue information were presented, and either information could have caused the reported action prime effects. This point is important given the suggestion of Liepelt et al. (2008) that both action kinematics and the representation of goals could contribute to the action priming effects. The fact that the priming was specific to matched action kinematics and matched action goals independently in the ACTION condition here illustrates this bi-determination of motor representations. This might suggest that goal attribution and kinematic priming use independent cue information from the observed action, perhaps implying that they involve two independent cognitive processes that co-occur in parallel. This rationale is consistent with proposals suggesting that observed action representation do not solely rely on kinematic matching, but also require top down goal attribution (Jacob and Jeannerod, 2005; de Lange et al., 2008). In this sense, our results also suggest that the common language between perception and action could vary regarding a degree of abstraction, ranging from a very close representation of the action (kinematics-related) to a more global form of representation (goal-related).

However, as mentioned above, the combined presentation of action and gaze (in the FULL condition) elicited a more fine-grained profile of priming for the time to peak grip aperture. It seems that in this later kinematic component, goal-related priming and kinematic-related priming operated complementarily. In other words, similar goals led to either facilitated action execution if observers’ hand trajectories matched that of the agent, or slowed action execution if there was a mismatch between observed kinematics and executed ones (see Ondobaka et al., 2012 for similar findings). These authors stated that when an agent’s action intention is relevant for the observer’s action execution, the kinematic-related priming is moderated by top–down goal ascription. We suggest that this is due to the perceived intentional value conveyed by an agent reaching for a target while his attention is directed toward the target of his reach. Jellema et al. (2000) described a population of cells in the anterior part of the superior temporal sulcus (aSTS) that responds preferentially to observed reaching action when the agent pays attention to the target of reach, compared to when attention is made elsewhere. According to the authors, eye-gaze in addition to an action would convey useful information to interpret the action as intentional. Under this interpretation, a correspondence between the agent’s direction of attention and reaching action would refine the observers’ motor representation to match the intention of the agent. Perceived eye-gaze direction could constitute a cue that allows the observers’ motor system to distinguish different motor programs aiming for the same goal. This stronger visuo-motor congruency would explain how and why actions with similar goals and different kinematics produced competitive motor responses in the FULL condition.

## Conclusion

In this study, we showed that agent’s gaze and action differentially, but complementarily contributed to an early representation of the action goal. We suggest that once the goal representation is understood by the observer’s motor system, the diversity of the visual cues available influenced the level of abstraction of the motor representation elicited. We showed that action cues permitted goal-related priming and kinematic-related priming independently, whereas combined gaze and action information triggered a more refined representation illustrating a specific intended action kinematics toward the selected goal. In this case, observers appeared engaged in a communicative link with the agent, maybe through the establishment of joint attention. This permitted for the elicitation of richer motor representations, probably indicating the understanding of the observed motor intention.

## Conflict of Interest Statement

The authors declare that the research was conducted in the absence of any commercial or financial relationships that could be construed as a potential conflict of interest.

## Footnotes

1 Summary tables of all of the results are given in the supplementary materials.
