# Supplementary material for: Different but complementary roles of action and gaze in action observation priming: Insights from eye- and motion-tracking measures
Source: Front Psychol. 2015 May 5;6:569. doi: 10.3389/fpsyg.2015.00569 (PMC4419854; doi:10.3389/fpsyg.2015.00569)
Supplement: Supplementary file 1 [file Table_1.DOCX]

***Table 1*** *: The following table summarizes the F values, p-values and effect sizes for the main effects and interactions for the ANOVAs testing the three factors of observation condition (Condition), object congruency (Object) and spatial congruency (Spatial) for the three motion-tracking dependent variables* (* p< ,05; ** p< ,01; *** p< ,005)*.*

**Dependent variables.**

***F*** ***p. η²_p_***

**Reaction time**

Condition 47.37 0.000 *** 0.72

Spatial 5.87 0.026 * 0.25

Object 0.59 0.453 0.03

Condition*Spatial 2.34 0.111 0.12

Condition*Object 5.87 0.006 ** 0.25

Spatial*Object 2.16 0.159 0.11

Condition*Spatial*Object 1.36 0.269 0.07

**Time to peak velocity**

Condition 1.83 0.175 0.09

Spatial 5.16 0.036 * 0.22

Object 0.12 0.736 0.01 Condition*Spatial 0.10 0.903 0.01

Condition*Objet 2.46 0.099 0.12

Spatial*Objet 0.15 0.708 0.01

Condition*Spatial*Object 3.36 0.046 * 0.16

**Time to peak grip aperture**

Condition 3.11 0.057 0.15

Spatial 5.43 0.032 * 0.23

Object 0.12 0.729 0.01 Condition*Spatial 3.27 0.049 * 0.15

Condition*Objet 5.73 0.007 ** 0.24

Spatial*Objet 0.37 0.549 0.02

Condition*Spatial*Object 7.72 0.002 *** 0.30
