# Supplementary material for: Different but complementary roles of action and gaze in action observation priming: Insights from eye- and motion-tracking measures
Source: Front Psychol. 2015 May 5;6:569. doi: 10.3389/fpsyg.2015.00569 (PMC4419854; doi:10.3389/fpsyg.2015.00569)
Supplement: Supplementary file 3 [file Table_3.DOCX]

***Table 3*** *: The following table summarizes the F values, p-values and effect sizes for the main effects and interactions for the ANOVAs testing the factors of object congruency (Object) and spatial congruency (Spatial) for each observation condition for the time to peak grip aperture dependent variable* (* p< ,05; ** p< ,01; *** p< ,005)*.*

**TIME TO PEAK GRIP APERTURE.**

***F*** ***p. η²_p_***

**Action condition**

Spatial 1.46 0.242 0.07

Object 14.96 0.001 *** 0.45

Spatial*Object 2.16 0.16 0.11

**Full condition**

Spatial 8.73 0.008 ** 0.33

Object 0.52 0.479 0.03

Spatial*Objet 5.28 0.034 * 0.23

**Gaze condition**

Spatial 0.45 0.512 0.02

Object 1.97 0.178 0.10

Spatial*Objet 7.23 0.015 * 0.29
